# Residential immersive life skills programs for youth with disabilities: a case study of youth developmental trajectories of personal growth and caregiver perspectives

**DOI:** 10.1186/s12887-019-1793-z

**Published:** 2019-11-06

**Authors:** Shauna Kingsnorth, Alanna E. F. Rudzik, Gillian King, Amy C. McPherson

**Affiliations:** 1 Bloorview Research Institute, Holland Bloorview Kids Rehabilitation Hospital, 150 Kilgour Road, Toronto, Ontario M4G 1R8 Canada; 2 Department of Occupational Science and Occupational Therapy, University of Toronto, Toronto, Canada; 3 Rehabilitation Sciences Institute, University of Toronto, Toronto, ON Canada; 4 Department of Anthropology, SUNY Oneonta, Oneonta, NY USA; 5 Dalla Lana School of Public Health, University of Toronto, Toronto, ON Canada

**Keywords:** Adolescence, Young adult, Autonomy, Experiential learning, Independence, Physical disability, Problem-based learning, Transition

## Abstract

**Background:**

Professional support in pediatric and rehabilitation care environments has been recommended as a means to build youth competence in life skills during their transition to adulthood. Life skills are the essential psychosocial competencies and interpersonal skills needed to manage one’s life. Residential immersive life skills (RILS) programs offer youth with physical disabilities enriched learning environments to acquire these skills. This study explored trajectories of personal growth in life skills and positive psychological outcomes among youth participating in a RILS program and related caregiver perspectives.

**Method:**

Delivered by a multidisciplinary healthcare team, *The Independence Program* is an intensive summer program housed in a college residence that provides realistic experiences of living away from home for small groups of youth between 17 and 21 years of age who have congenital and/or acquired physical disabilities. Using a longitudinal case study and qualitative descriptive design, four youth and their parents/guardians participated in semi-structured interviews prior to, and then 1 month, and 3 to 4 months after the program. A conventional content analysis yielded chronological narratives for each youth and caregiver dyad of their experiences, perceptions and outcomes over time. These narratives were further summarized using a ‘line of development’ perspective to describe individual developmental trajectories of personal growth.

**Results:**

All four of the youth returned from the program with positive reports about the new life skills acquired and new behaviours they engaged in. These positive reports generally continued post-program, albeit with differing trajectories unique to each youth and varying levels of congruence with their caregivers’ readiness to support, accommodate and facilitate these changes. Caregivers differed in their capacity to shift in their parenting role to support consolidation of youth life skill competencies following program participation.

**Conclusions:**

RILS programs can be transformative. Varied youth trajectories identified significant personal growth through enhanced self-determination, self-efficacy and self-advocacy. Congruence in youth and caregiver perceptions of post-program changes was an important transactional factor. Professional support addressing caregiver needs may be beneficial to facilitate developmentally appropriate shifts in parenting roles. This shift is central to a model of shared management whereby adolescents take on greater responsibility for their own care and life choices.

## Background

Over the past two decades, there has been a significant shift in recognizing emerging adulthood as a distinct developmental period [1, 2]. During their late teens and early 20s, young people begin to plan and take action for a future that typically includes continued education, advanced work and career paths, and enhanced social, intimate and sexual relationships, among other life areas [3, 4]. Research has flourished on the timing, nature and sequence of activities and changes that adolescents experience in pursuit of these and other markers of adulthood [5–7].

While much of the available literature has focused on the developmental trajectories of typically developing youth during this period [2, 4], there is growing attention being placed on the experiences of young people with disabilities [1, 3, 8]. Though they frequently share the same wishes and dreams for their future [3], many young people living with disability lag behind their peers in fulfilling traditional adult social roles [9, 10]. While many complex factors affect the person-environment interactions needed to attain these traditional markers of adulthood, several key barriers for youth with disabilities have been identified. Most notably, they experience starkly different early experiences in childhood and adolescence that facilitate the acquisition of essential skills and positive psychological outcomes (e.g. self-determination) [5, 11, 12]. Compounding these experiences, functional and/or cognitive limitations often mean that young people with disabilities may not have the same expectations or opportunities for choice-making and problem solving through equivalent interactions [13]. Moreover, people may perceive them as more vulnerable and at risk than their typically developing peers, and continue to act in protective ways that impede the development of autonomy within periods of adolescence and young adulthood [8, 13].

Professional support in pediatric and rehabilitation care environments has been recommended as a means to build youth competence in skills essential to personal growth during this period of emerging adulthood [8, 11, 13, 14]. As Turnbull & Turnbull [15] outline: “[people with disabilities] need training designed to increase their capacities to make, communicate, implement, and evaluate their own life choices” (p.117). Enriched environments, such as those afforded by residential immersive life skills (RILS) programs, are one mechanism to achieve these aims. RILS programs offer targeted opportunities for acquiring life skills through situated learning and group immersion experiences among peers within a positive and youth-oriented environment [16–20]. As defined by the World Health Organization [21], “life skills are psychosocial competencies and interpersonal skills that help people make informed decisions, solve problems, think critically and creatively, communicate effectively, build healthy relationships, empathize with others, and cope with managing their lives in a healthy and productive manner” (p.8).

Evaluative data on life skills programs for youth with disabilities are emerging. Broadly, investigations of life skills programs with varied levels of immersion have demonstrated positive outcomes [13, 16, 22]. In a programmatic series of past studies by our research team, we have retrospectively examined perceived program impact among alumni of three different RILS programs using survey and interview methodologies [16, 19], and investigated provider perceptions of key program features and experiential benefits [23, 24]; thus building on prior work examining short-term impact [14, 18]. Across this body of work, participation in RILS programs has been described as a ‘transformative experience’ reflecting accelerated personal growth [16–19].

This compelling descriptor raises a number of interesting questions as a function of participation in a RILS program – most notably, what types of personal growth contribute to this experience? When are these changes happening and how are they experienced across individuals? And what roles do parents play in supporting or inhibiting consolidation, maintenance, and generalizability of these changes? To date, little research has sought to understand more richly how experientially-based RILS programs influence acquisition of life skills and positive psychological outcomes central to the period of emerging adulthood among youth with disabilities [8, 12]. The current study begins to address this gap by exploring perceptions of personal growth due to RILS program participation via youth developmental trajectories, and youth and caregiver perspectives.

## Method

This study reports on the qualitative arm of a prospective, mixed-method observational pilot study designed to assess the feasibility and utility of selected qualitative and quantitative methods prior to their application to a larger sample [17, 25]. Feasibility-related findings for the quantitative arm addressing program characteristics (opportunities and intervention strategies) in a range of activity settings via validated self-report measures and observer-completed measures have previously been published [17, 26]. The qualitative arm extended prior work via prospective longitudinal data collection and addition of caregiver perspectives [18]. A qualitative descriptive design methodology was employed to gather rich descriptions of experiences, from the unique viewpoint of the participants [27].

Taking an exploratory approach, our aim was to begin to investigate trajectories of personal growth among youth participating in a RILS program and their caregivers’ perspectives of changes that may (or may not) have occurred. For the purposes of this paper, personal growth was defined as the process of redefining oneself through maturation and self-directed change, resulting in enhanced self-determination, self-efficacy and self-advocacy competencies [28, 29]. To describe trajectories of personal growth, we applied a ‘line of development’ perspective to capture chronological changes in an individual’s ‘path’ [12, 30]. A longitudinal case study design was used to gather data from youth and their caregivers, allowing us to capture details of their interactions and home contexts relevant to the acquisition of life skills and psychological outcomes at different time-points [31]. Definitions of these positive psychological outcomes associated with personal growth can be found in Table 1 [28, 32–35].

**Table 1:** Positive psychological outcomes associated with personal growth

Outcomes	Definition
Self-determination	Self-determination refers to an individual’s ability to make choices and exert control over one’s life [32]. Self-determination helps individuals meet basic needs related to autonomy (control over one’s life), competence (mastery) and relatedness (close relationships) [28].
Self-efficacy	Central to self-determination is self-efficacy, which refers to an individual’s beliefs about his/her ability to cope with challenges and be successful in specific situations [33].
Self-advocacy	Associated with self-determination, self-advocacy refers to “an individual’s ability to effectively communicate, convey, negotiate or assert [one’s] own interest, desires, needs and rights” (p.1) [34].

### Participants and recruitment

A purposive cohort of eight youth attending a RILS program called *The Independence Program* (TIP) were approached about participation in the research. A target of 50% recruitment was set as three to five participants are recommended for a case study approach [36]. Caregivers included parents and other family members responsible for youth care. Ethics approval was granted from Holland Bloorview Kids Rehabilitation Hospital, Toronto, Canada. Initial contact was made by a service provider familiar to the youth who explained the nature of the research. A research assistant then met with interested youth and their caregivers to further explain the study, obtain written consent, and schedule the interviews. All participants provided informed consent prior to participation.

### Intervention

TIP is a 3-week intensive program housed in a college residence that provides realistic experiences of ‘living away from home’ for small groups of youth between the ages of 17 and 21 who have congenital and/or acquired physical disabilities (e.g., cerebral palsy, spina bifida, brain injury). This annual summer program is offered by a large pediatric rehabilitation hospital and delivered by a multidisciplinary healthcare team (i.e., occupational therapy, physical therapy, nursing, life skills and therapeutic recreation). Attendant care is available to assist with activities of daily living (such as personal grooming, dressing or transferring) for participants who require additional support. During the day, youth take part in a formal curriculum that includes sessions on time management and organization, financial literacy, travel planning and independent mobility, grocery shopping and meal preparation, among other topics; a daily schedule exemplar can be found in King et al. [23]. During the evening, youth engage in less structured learning and community building through organized and/or informal social activities. Details of this long-standing program and its theoretical foundation of social learning, experiential learning, and self-determination theory have previously been described in the literature [17, 18, 23, 24, 26].

Broadly, the program targets youth who are motivated to participate and interested in increasing their independence. To be eligible, youth must be cognitively able to identify a transition-related goal; have the capacity to communicate personal care needs; must not have behavioral issues that would impact participation in a group-based learning environment; and be deemed medically stable by their family physician. Applicants submit a written application and participate in a phone interview to assess their fit with program demands. In addition to the program fee, participants are encouraged to raise funds to cover a preset spending allowance and have a personal debit card for budgeting and other program activities.

### Data collection

In-depth, open-ended interviews were conducted at three time points: 1 to 3 days prior to the beginning of the program (pre-TIP), 1 month after the end of the program (post-TIP), and 3 to 4 months after the end of the program (follow-up-TIP). Caregivers and youth were interviewed separately at each time point, either in person or by phone. All interviews were conducted by a trained research assistant with extensive interview experience using semi-structured interview guides.

The interview guides consisted of broad questions, allowing the interviewer to probe for further details within each area of discussion [37]. Table 2 provides examples of questions posed. In the pre-TIP interviews, we probed for specific information about youth and caregiver expectations going into the program. In the post-TIP and follow-up-TIP interviews, we reviewed the earlier identified expectations and probed for examples and stories concerning how these were being met, as well as future goals and aspirations. Finally, we asked about the maintenance and generalizability of the skills gained by youth, and the role of caregivers/family in helping the youth to maintain any perceived gains upon returning home.

**Table 2:** Select examples of open-ended questions from the youth interview guides. Questions for caregivers were modified to reflect their perspectives

Timepoint	Youth
Pre-TIP	*I’d like to know a bit about how you decided to take part in TIP?* *How did you hear about it?* *Why did you think it might be a good program to take part in?* *What was the main reason you applied to TIP?* *Did you make the decision on your own or with other people?* *Why do you think Mom/Dad/Other wanted you to take part (if appropriate)?* *What do you hope you to get from attending TIP?* *What do you think will happen in the program?* *Are there things you are most excited about?* *Are there things you are worried about?* *What do you think will be the most challenging part of the program? What do you think will be the easiest part of the program?* *What do you think a successful TIP experience will look like?* *What would you like to happen during TIP?* *What would have to happen (or what would you have to learn) during TIP for it to be a really good experience?* *What do you think your Mom/Dad/Other want you to gain out of attending TIP?*
Post-TIP and Follow-up-TIP	*Tell me about your experience of taking part in TIP?* *What was your greatest success? What was your greatest challenge?* *What were the most difficult parts? What were the easiest parts?* *What was the outcome in terms of the goals you set at the beginning of the program and your plans for the future?* *Do you think TIP influenced you?* *Tell me about a story – good or bad – that show how TIP affected you?* *For each story:* *What did you realize? When did you realize it? How did you realize it?* *Is that something you could have done before the program?* *What was it about the program that made the difference?* *Tell me about what happened when you got home?* *Did anything change about your expectations of yourself? Others?* *Did other people have different expectations of you?* *Is there something you are now doing that you didn’t do before you took part?* *Is there something you are going to try (or have already tried) for the first time? How easy or difficult do you think it will be to keep doing these things?* *Thinking about the program and the things you are doing now, would you do anything differently during the program if you could do it again?* *After the program? Is there anything that you wished you could have practiced or learned?* *What do you think is the most important thing you learned at TIP? About yourself? About others? Why do you think so?* *If you had a chance to do the program over again, would you? Why or why not?*

All interviews were digitally recorded and professionally transcribed verbatim. Transcripts were checked for accuracy and all identifying information was removed prior to analysis, with pseudonyms assigned to each participant. To minimize risk of participant identification from a small program, only basic demographic information was collected for youth, including gender, type of disability, level of mobility, size of their community of residence and health status. Caregiver demographic information was limited to gender*.*

### Data analysis

A conventional content analysis was used [38], appropriate to qualitative descriptive studies where “straight descriptions of phenomena are desired” (p. 334) [39]. Employing a paired approach, the transcripts for each youth and caregiver were viewed as a dyad or case [31, 40]. Drawing upon Braun and Clarke’s analytic framework [41], transcripts from three dyads were first individually reviewed by three of the authors, with backgrounds in social, health and developmental psychology. Key points were noted that related to expectations, motivations, experiences, and perceptions of the program, as well as impact on life skills and positive psychological outcomes. The group met to compare notes, refining and organizing these key points into initial codes; early impressions of emerging patterns in trajectories in personal growth were also discussed [41].

From this preliminary stage, the second author, an anthropologist, reviewed the transcripts of all four dyads in full, applying these codes and generating chronological narratives for each youth and caregiver. NVivo 8.0 was used to support data management and organization of codes and key quotes. These narratives were further reviewed and discussed by the first and second authors, and subsequently summarized as chronological trajectories, using a descriptive ‘line of development’ perspective to arrange the data [30]. Coded narratives and trajectory summaries were then presented back to the remaining two authors and discussed by the full group to establish confirmability of the findings [41, 42]. This approach was beneficial in two ways. First, it afforded a dyadic aspect to compare and contrast youth and caregiver expectations, motivations, experiences and perceptions of the program, and its outcomes at the same time points. Second, it allowed the creation of a detailed picture of the impact of the RILS program on individual trajectories [27].

## Results

Target recruitment was achieved with four youth-caregiver dyads from the larger cohort of eight TIP participants taking part. One youth was female and the other three were male; three caregivers were female and one was male. Diagnoses included cerebral palsy (2), acquired brain injury (1) and muscular dystrophy (1). Perceptions of health status were positive across all four youth, with ratings of good (1), very good (2) and excellent (1). Three of the four participants required the use of a wheelchair or other aid to facilitate mobility. Two youth were from major urban centres, one was from a smaller urban centre and one lived in a rural community.

Average interview lengths for youth were 25 min, 78 min and 60 min for pre-TIP, post-TIP and follow-up-TIP, respectively; caregiver interviews were somewhat shorter in duration at 24 min, 46 min and 38 min for the three time points. In keeping with the case study design, changes over time and the nature of these changes for each youth-caregiver dyad are described individually in participant narratives. These narratives are then summarized as a case using a ‘line of development’ perspective to characterize individual trajectories of personal growth [31].

### Case study narratives

#### Dyad #1: JP (youth) and Terra (caregiver)

JP’s motivation to attend TIP was *‘to become more independent … and not rely on others’*. In addition to specific independence-related tasks, he said that he was most excited about ‘*meeting new people*’. Terra hoped that JP would ‘*come out and really interact and ‘make friends*’. Terra felt that ‘*It’s time for [JP] to learn to be really independent … he’s at the transition*’. Although Terra was the one who attended the TIP information session and collected the application form, she encouraged JP to take more responsibility as they completed the application: ‘*I said “You do it and then you learn how to answer*”’. While Terra wanted JP to do tasks around the house, she described herself and her husband as overprotective and not comfortable helping him learn to cook, for example. At the same time, she expressed the desire for JP to ‘*do something by himself without me telling him what to do*’ and talked about the possibility of a future where JP would live in a college residence, away from home.

In the post-TIP interview, JP spoke about the new level of independence that he experienced at TIP compared to other, non-immersive life skills programs he had previously taken part in. ‘*Other camps I’ve been to, they helped me along the way. At TIP you do somewhat everything yourself*’. In contrast to Terra’s statement during her pre-TIP interview that JP didn’t like to go out on his own, JP reported enjoying the freedom and challenge offered at TIP whereby participants were encouraged to plan outings: ‘*Like for me I don’t go out that much and it’s fun to go out sometimes. And getting lost and finding a way back*’. He expressed enthusiasm for having tried things that were scary or challenging.

In talking about his life 1 month post-TIP, the main change that JP described was that he was now more talkative and sociable than previously. Terra, though, expressed disappointment in the lack of positive change that JP had shown since returning home, for example, ‘*he hasn’t done much … like chores around, he hasn’t done anything*’; ‘*socialization, he hasn’t really made that many friends yet*’; and ‘*he will still look at [me] for guidance*’. However, when JP did make bids for autonomy, Terra reported that she had responded supportively. In particular, she noted that JP had been self-advocating at school: ‘*He said “I don’t want to do [program]” … so he went and spoke to the teacher and then he went to see the counsellor*’. In the past, she noted that JP would have asked her to contact his teacher. Terra’s response was to encourage him to take even greater responsibility: ‘*I said “You go and speak to [teacher] … I’m not doing anything. You are an adult now, go and speak to them. Find things yourself” … Before, yes, I would speak up for him more, yeah. He would look at me for things*’. Despite these positive signs, 1 month post-TIP, Terra still expressed doubts about JP’s capabilities, saying ‘*I’m still looking at, what can JP exactly do?*’

By the time of the follow-up interview 3 months after the program, JP’s ability to self-advocate had increased substantially. He had decided for himself to make changes to the support that he received at school. He proudly stated that he was getting to classes ‘*all by myself, on time*’. Terra’s impression of the change in JP by this point was considerably more positive. She said ‘*He’s becoming much better now. He’s independent*’ and provided examples from school and accessible transit booking. In addition to those specific tasks, she brought up his ability to problem solve when in the past he would have remained passive. ‘*He’s able to look and say “Oh, that’s the issue here, that’s a problem I need to solve, I’ll go to the teacher and I’ll tell the teacher” … [Before TIP] he would just sit there and wait*’. She ascribed the change in JP’s self-efficacy to his experience at TIP: ‘*In TIP, I think with a lot of coaching, he felt more secure. He felt “If I can work at it, if I do what they teach me, I should be able to do it”. He feels a little bit more confident … initially he didn’t show it but for a few months, now he’s been showing it’.*

Her response to JP’s increased independence grew increasingly more supportive and less protective across the three time-points. One month after the program, Terra talked about how, if JP wanted to make a hot drink after school, she would give JP the hot water because she and her husband were nervous allowing him to use the electric kettle. By 3 months post-program, he had begun using the kettle by himself. Terra identified her own role in helping JP build on his time at TIP, saying: ‘*I learned to be less of a mother hen … [not] to say “JP did you do this? JP did you do that” … I said “Okay, when you’re ready, just let me know, I’m not telling you what to do”*’.

#### Trajectory summary

JP’s line of development can be described as an accelerating positive curve. In the beginning, changes reflected steady growth in mastery and consolidation of skills across the program to 1 month post. This was followed by a more dramatic increase from 1 to 3 months. During this latter window, his caregiver was increasingly more aware of her role, stepping back and affording him more opportunities for independence.

#### Dyad #2: Jacob (youth) and Dustin (caregiver)

Jacob stated that his motivation for attending the program was ‘*to learn how to live on my own*’ before starting college and to have more confidence in himself. He was unsure what his experience at the program would be like and shared that the most challenging part would be ‘*being away from my parents*’. His father, Dustin, confirmed that he and Jacob’s mother were the main instigators of Jacob’s application to the program. Dustin’s main desire was that Jacob would be exposed to ‘*real world*’ situations to ‘*see what he could do away from Mom and Dad*’. He hoped Jacob would benefit from the social exposure provided by the program and develop some self-advocacy skills. Specifically, he wanted Jacob to ‘*have different people look after him and be comfortable [with that]*’. Dustin was not optimistic, however: ‘*it all depends on, like, if he’s around a core group of kids he likes, or just comfortable with, he’ll excel. But … depending who’s around, he might not. I don’t really know*’.

At the post-TIP interview, Jacob was very positive about his experience at TIP and identified greater independence and autonomy. ‘*I didn’t want to come home. I liked [the program] … being on my own*’. He enjoyed ‘*getting to go out and do stuff that [I] wanted to do*’. He found that the program pushed him to try different things; even if it was something he didn’t like to do or was unsure about, he often found that it was easier than he had expected. When asked about any changes since the program, Jacob mentioned feeling ‘*more positively about people and talking to them*’. Dustin confirmed that Jacob’s communication skills had improved since the program, including with his immediate family.

Dustin described the program as a ‘*life-changing experience*’ for Jacob, saying ‘*When Jacob came out, he came out whole, he came out bubbly … he just seemed to be an outgoing person*’. He was pleasantly surprised that his son had had good camaraderie with his roommates saying ‘*it was kind of borderline*’ whether he would: ‘*It didn’t really surprise me, but it does surprise me*’. Dustin shared some of the higher-level skills that Jacob had built at TIP, like self-determination: *‘[TIP] wasn’t “do this, do that”. It was just “oh, what do you want to do now?”’*. He had a very positive impression of the skills that Jacob talked about having used during the program; perhaps as a result, Dustin said, ‘*I learned that he is capable of doing it, living on his own … I wouldn’t have thought it a year ago*’. Following the program, Dustin facilitated Jacob’s desire for increased independence. On a family vacation shortly after TIP, he said to Jacob: “*‘Okay, I’m driving, you’re guiding” … If I was going to miss a turn, [I was] going to miss a turn … because he was guiding … It kind of put him more in the driver’s seat so to speak*’.

By the follow-up interview, the increases in Jacob’s communication and social skills translated into increased self-efficacy and self-advocacy. Jacob gave an example of following up on a financial matter for himself: ‘*if I have an issue I call it in instead of asking [my parents] … I called and did it all on my own*’. Dustin spontaneously mentioned that Jacob had handled the incident on his own, which *‘would have been totally the opposite last year’*. Dustin’s understanding of the meaning of ‘independence’ also changed as a result of Jacob’s exposure to TIP. ‘*I was thinking [that to be independent] … you had to do everything yourself … .the way they explained it was that independence is, if you need help you ask for it.*’

Dustin’s horizons for his son had broadened as a result of Jacob’s increased self-efficacy post-TIP. ‘*He knows he can do this so it’s just, now let’s move forward with life, get to college and so on*’. Dustin felt that Jacob was happier following the program: ‘*He doesn’t give you the impression that he’s depressed or anything like that*’; *‘[he doesn’t] look down or out or unhappy. You always feel like [he’s] got life in [him]*’. Dustin was supportive of his son’s new behaviours and said that he and his wife were now ‘*more comfortable with him being [at college] without us*’.

#### Trajectory summary

Jacob demonstrated a positive linear trajectory via his experience at TIP and subsequent return home. Acknowledging Dustin’s characterization of the experience as life changing, Jacob’s line of development may be described as steady, increasing from the outset with his self-reported mastery and continuing in this path. Dustin enthusiastically embraced Jacob’s new skills, providing significant opportunity and encouragement towards self-mastery.

#### Dyad #3: Beth (youth) and Susie (caregiver)

In the pre-TIP interview, Beth spoke about plans for the future and living on her own in residence at a postsecondary institution. She contrasted her perception with how she thought her parents perceived her, saying ‘*they may say “You’re not ready for that” but I say “Sure I am, I can do this and do that, I’ll be fine!”*’ She planned to use her attendance at TIP to convince her parents of her ability to live on her own. Beth was motivated ‘*to see what I’m capable of*’ and identify her weaknesses as well as her strengths.

Susie also had a positive view of TIP and felt it would provide an opportunity for Beth to develop more accurate self-perceptions: ‘*either she will go there and she will learn skills to help her to be independent, or...the other outcome could be...recognize some of her real challenges*’. Where Beth believed her parents were too focused on her learning day-to-day tasks, like how to keep her room clean, Susie drew a connection between those day-to-day skills and the larger life skills needed for functioning in the world as an adult ‘*The truth is … it’s the same life skill that you can keep your things organized and then you keep yourself organized*’.

During the post-TIP interview, Beth described a largely positive experience at the program, noting that it exceeded her expectations in providing a challenging and stimulating environment. While she experienced several major challenges and moments of anxiety, those moments turned into successes as she accomplished what she set out to do. After returning home, Beth said: ‘*I feel maybe I expect more from myself...I’ve learned so much more about how I could do things for myself, I don’t really expect people to do as much as I expected them to do before*’. Her sense of self-efficacy had increased, which led to increased willingness to try new things ‘*I think just being able to have the confidence to do things and be able to implement a plan*’. Part of Beth’s increased self-efficacy arose from shifting her definition of independence towards interdependence as a result of the program: ‘*I’m more aware now that you can get people’s help and still be independent*’.

Returning home from the program, Beth felt that her parents weren’t keeping up with the pace of her change: ‘*sometimes I feel like my parents don’t know the extent of what I did at TIP...I don’t think they know what I was capable of’*. Beth regretted that she had not followed the advice of TIP staff to have a conversation with her parents as soon as she returned home. In contrast to Beth’s view of her parents’ limited awareness, Susie noted increased self-determination and self-advocacy post-TIP, which she described as ‘*a dramatic change in terms of the independence levels*’. In particular, Susie was impressed with the way Beth had taken responsibility for all of her college-related decisions, including dealing with disability services, choosing courses and buying her books. She attributed these changes to Beth’s participation in TIP: ‘*I think if she didn’t go to TIP she would have been content in the way we’ve operated over all the years*’. Susie also acknowledged that her hopes for TIP had been more day-to-day, rather than focused on the higher order life skills: ‘*My expectation was that [after TIP] she would have been able to manage her room...I wasn’t looking for the bigger things about, well, managing your own life*’.

By the follow-up interview, both Beth’s social participation and her horizons for the future had broadened. With increased confidence in navigation, she joined committees and attended meetings at her former treatment centre. The increased self-efficacy that she had developed expanded her ideas of where she might attend university to schools outside of her home city. The transferability of the skills she learned at TIP was central to this: ‘*if I can do three weeks...I can just use those skills long term, right? For months and years...Now I know, yeah, I can handle living by myself*’. Susie was proud of the independence and self-determination that Beth showed, saying ‘*she has taken serious responsibility as regards, like managing her education and going to school and doing her homework and keeping on task and doing her social things*’.

Beth also described moving into a more typical young adult relationship with her parents: ‘*I’m out of the house a lot more than I’m in the house and even now it’s a weird adjustment for them because I went from always here to not*’. Despite this, Beth felt limited in her relationship with her family: ‘*I honestly feel like they don’t think of me any different from a high school kid, you know? And that’s rough, that’s just hard*’. Susie encouraged Beth’s independence by holding back on tasks that she would previously have done for her: ‘*I used to remind her every night before we’d go to bed--okay please charge your [phone and tablet]....I don’t do any of that now*’. This change in behaviour on Susie’s part was encouraged by TIP program staff to foster a more ‘*typical teen*’ experience: ‘*the staff has really pushed me in terms of letting go and letting her be*’.

Overall, the program caused a shift towards optimism in Susie’s view of Beth: ‘*I see it more now as yes, she can be independent, and yes, she will get an education, yes, she will be able to find a job and she will be able to take care of herself. Like, I can see that more in reality now. [Before TIP] it was kind of like debatable. I, I wasn’t sure*’.

#### Trajectory summary

Beth’s line of development can be described as stepwise. Though moving in a positive direction, she experienced fluctuations during her initial TIP experience before stabilizing in an upward direction by program end. This positive trend continued in the first month post-TIP, followed by a steeper increase over the course of the remaining 3 to 4 month follow-up period. Susie’s perspectives appeared to mirror Beth’s trajectory with some significant shifts in the dynamics of their relationship arising.

#### Dyad #4: Lucas (youth) and Celeste (caregiver)

In his pre-TIP interview, Lucas launched into his reasons for wanting to attend TIP before even being asked: ‘*I’m at that age now where I gotta start taking more responsibilities of my own things*’. He was proactive about the decision to participate and had attended the information session himself. Lucas felt that success would be ‘*to be the most independent as possible*’ but he had a realistic outlook, saying ‘*I know it won’t happen overnight … but I just want to get that little bit more independent*’.

Celeste hoped that TIP would be ‘*a little test for [Lucas], to see how he can function without Mom and Dad*’. At home, Celeste was very closely involved with Lucas’s self-care, whereas *‘[at TIP] Mom won’t be there to check how he’s brushed his teeth or you know how he’s dressed*’. Celeste wanted Lucas to become ‘*more aware of the stuff that’s done for him*’. She was less vocal about him learning to do the tasks for himself, noting that she and her husband do not take Lucas to the grocery store or teach him to shop for meals because of time constraints. In sharp contrast with Lucas, Celeste’s expectations for TIP were not high: ‘*I mean he’s going to learn something, how can he learn it in less than three weeks?*’ She did not see Lucas as likely to thrive in the program setting ‘*I’m worried for him. Lucas, he’s got a lot of strength, but he also has a lot of needs … if he’s asked to do something on his own, I don’t know*’.

At the post-TIP interview, Lucas was enthusiastic about the impact of TIP: ‘*I’m so glad I did it, you know? It taught me a lot of life skills, let alone about myself, right?*’ Describing how he felt before the program, Lucas shared: ‘*I was thinking the worst things possible [about how I would cope]*’ but afterwards noted that ‘*confidence is the biggest thing … if you have confidence and if you believe you can do anything, don’t worry, you can*’. Reflecting on returning home, Lucas also said: ‘*I want to do more of the things on my own … I always chose what [leisure activities] I wanted to do but now I think I’m ready … to take more of a lead in that role*’. However, Lucas contrasted the situation at TIP with home *‘[At TIP] I could do everything at my own pace and didn’t have to worry about deadlines … I wish I could do that more now, but … you’re working against time*’.

In contrast to Lucas, Celeste was more reserved in her reflections on changes post-program. While she noted that he was more aware, *‘ … three weeks, of course is not long enough, right? But at least it got the wheels turning and made him think about his future and think about, you know, maybe I can live independently with attendant help*’; Celeste’s response to Lucas seeking to take on more tasks was not entirely supportive. At the post-TIP interview, she said: ‘*He volunteered “Mom, do you want me to take the laundry out of the machine?” That was cute … I thought that was cute*’. Rather than working to nurture his emerging skills, her perspective on independence was an all-or-none approach: *‘ … what am I going to expect, miracles? He’s going to cook my dinner? He’s not*’. Though Lucas felt that he’d learned a lot about his capacities from the program, when asked if she had noticed changes in Lucas, Celeste replied: ‘*I don’t think so. I haven’t noticed any change … I think I know him very well*’, further commenting, ‘*I’m still doing everything I did before he left*’.

By the follow-up interview, Lucas’ enthusiasm about his gains from TIP had faded somewhat: ‘*I guess overall it was a good experience but … I just wanted to figure out better ways to do … a lot of these things in my daily life*’. While Lucas identified that TIP helped him begin ‘ *… to start the process to take more initiative on my own*’, he had not been able to incorporate all of the skills learned into his home life. Describing meal preparation, Lucas said ‘*by the end of [TIP] I was able to cook with an attendant’s help, but … it’s not realistic for me … how am I going to really put that into practice at home in my daily life?*’ Despite challenges in his home environment, Lucas felt he successfully met his goal of attending post-secondary school, sharing: ‘*I’m happy about that I’m … handling college … college in a good and positive manner*’.

In contrast, Celeste’s enthusiasm for Lucas’ skills was just beginning to grow, sharing that he was now making arrangements for transportation to school, gradually taking over from his parents: ‘ *… which is a big help … and just the fact that he’s going there independently...we’re so proud of him*’. Celeste’s reflections on Lucas’ capabilities were sometimes mixed. She talked often about the burden of caring for Lucas and how he needed to learn to do things for himself. At the same time, she insisted that he was not capable of self-care, saying ‘*He cannot bathe himself. Cannot. He cannot get dressed independently*’ but later clarified, ‘*he’s always done that, but you know, he doesn’t do things correctly*’. These statements suggest that Celeste held a view of independence as one of physically doing all tasks without assistance: ‘*anything where he doesn’t need my help, I consider him independent in that area*’ and holding these activities to high standards: ‘*Well, he started watching me do it, and making sure, you know, I … I went over the, you know, everything he has to do not to make a mistake*.’ In other areas where Lucas might have practiced his new skills by helping around the house, Celeste maintained that it was not possible because ‘*We’re just too busy … when you’re limited with time, things have to get done*’.

#### Trajectory summary

Following TIP, Lucas was extremely positive, identifying many new skills and describing the program enthusiastically suggesting a fast rise. By the final interview, however, Lucas’ earlier upward trajectory began to plateau and he was notably downbeat. His line of development could be described as somewhat S-shaped; a pattern influenced by Celeste’s ambivalence towards his desire for greater independence and autonomy.

## Discussion

These case studies were drawn from data gathered from a sample of youth and their caregivers, following a RILS program. The chronological narratives demonstrated personal growth in the form of broadened horizons, shifting views on independence and autonomy, and enhanced competence and confidence in life skills and activities of daily living among participants and their caregivers. All four of the youth returned from their time at TIP with positive reports of new skills learned and new behaviours they engaged in, aligning with previous studies of RILS programs [14, 16, 19]. As examples, JP showed an accelerating increase in self-mastery post-program. Similarly, Jacob’s growth steadily increased in efforts towards self-determination over time. Beth also left the program feeling that she was capable of much more than she’d previously thought. Immediately following, Lucas was perhaps the most positive of the four youth and was looking forward to putting his new skills to use around the home.

These positive reports generally continued post-program, albeit with differing trajectories unique to each youth and varying levels of congruence with their caregivers’ readiness to support, accommodate and facilitate these changes. While JP, Jacob and Beth reported continued increases in their ability and willingness to make decisions for themselves, problem solve and cope with new situations as they arose, Lucas’s positive attitude noticeably shifted over time, influenced by family dynamics and current circumstances. This observation brings to bear greater consideration of how a young person is situated within their home context and the dynamic processes taking place in response to challenging life experiences and/or situations.

Application of a transactional framework is particularly apt for understanding processes during periods of transition such as emerging adulthood [12, 43]. The combined use of descriptive qualitative methodology and a longitudinal case study design afforded exploration of person-environment fit [31], offering insight into the individualized trajectories experienced. Namely, how youth use and build on skills discovered or developed at TIP, and how this changed over time within their situated contexts [43]. The inclusion of caregiver perspectives further shined a light on the dynamic adjustments taking place in relationships/roles between caregivers and youth post-program.

Differences in caregiver perception of youth’s competencies of newly acquired skills and willingness to facilitate their use emerged from the case studies. Where caregiver perceptions were congruent with youth self-perceptions, positive trajectories were noted with accelerating lines of development. Three of the four youth had caregivers who affirmed their nascent shifts in confidence and/or identity experienced early on and afforded continued opportunities to build on skills and behaviors acquired at TIP. Dustin, Terra and Susie all talked about making space for their youth to use their newly developed or strengthened skills. In the family trip example, Dustin facilitated Jacob’s new found desire for self-determination, tasking him with the responsibility of navigation. Post-program, Terra actively encouraged JP to take on increased responsibility, even when she was nervous or uncomfortable with things JP wanted to do for himself. Likewise, Susie felt it was appropriate for her to take a step back and let Beth take control of her actions and decisions. As caregivers, Terra and Susie both talked about refraining from reminding youth of tasks that needed to be completed, thus relinquishing control or responsibility to the youth and enabling natural consequences. This reflects the universal need for a shift of caregivers from a ‘manager’ to a ‘consultant’ role as youth mature [44, 45].

This qualitative longitudinal case study suggests that this shift is critical following youth experience with an intensive life skills program like TIP, to aid youth in skill consolidation, maintenance and generalizability. For some caregivers, the role change required for the young person to develop real independence and autonomy may feel uncomfortable (to the point of impossible) in the face of other, often pragmatic, constraints in life such as time and money. Caregivers’ own readiness to provide “room for more independence” can also vary (p.38) [8], and may be incongruent with the youth’s desires. As an example, the fourth youth, Lucas, had a very different experience upon returning home. Unlike the other caregivers, Celeste initially stated that she had not noticed any change in Lucas, which contrasted with his strong feelings of accomplishment post-program. Celeste also provided fewer examples of actively encouraging Lucas’ use of skills learned at TIP, noting that to do so was more time-consuming; yet opportunities to take some of the caring strains off from his parents were not prioritized. The contextual dynamics of this youth/caregiver dyad aligns with previous findings from independence related research where some parents are less positive than their youth about their emerging independence, leading to tensions in their relationships [45]. They may inadvertently emphasize the youth’s disability over their skills, which can lead to a situation of age-inappropriate dependency for the youth [8].

Youth were thus not the only benefactors of the accelerated learning environment and expertise of the multidisciplinary program staff. Initially, Susie had been focused on improving Beth’s day-to-day living skills. Following encouragement from TIP staff, she was able to facilitate the new, higher-order life skills that Beth developed through attending the program, by affording different opportunities that encouraged self-reflection. Dustin also believed that he had himself gained a new perspective from staff at TIP. In Dustin’s case, his understanding of the meaning of independence for his son shifted towards a model of ‘interdependence’, which allowed him to still provide Jacob with support, while acknowledging Jacob’s actions as fundamentally self-determined. Susie’s and Dustin’s comments suggest that there may be significant value in providing additional ‘formal’ support to parents and other family members, to enable necessary shifts in caregiver-child relationships/roles and long-standing patterns of behavior following intensive life skills programs [8, 11, 13, 14, 45].

### Strengths and considerations

The case study design afforded an in-depth look over time into youth and caregivers’ perceptions (experiences and expectations) prior to, during and post TIP, contributing to an understanding of individual variability in trajectories of personal growth that youth may undergo. Of note, the TIP cohort from which we drew focused on youth with a limited range of physical disabilities that may be relatively less severe than youth in other RILS programs. While the detail provided in the narratives aims to enhance transferability of the findings to youth who share similar environmental contexts and personal characteristics, others may experience very different trajectories. Also, as the sample included only one male caregiver and one female youth, gender differences cannot be examined. Future exploration is warranted to examine differences in readiness for change across gendered caregiving roles, as well as priorities for growth among youth themselves [45].

## Conclusion

Taken together, these narratives affirm that youth who attend RILS programs have the opportunity to develop new skills and confidence away from home. These programs can alter a young person’s developmental trajectory towards adult roles through ‘transformative experiences’ [12, 16, 19, 46]. Caregivers who embraced program-related changes among their youth can facilitate continued and long-lasting skill development, making the TIP experience (and other RILS programs) only the first step in an upward trajectory of personal growth [16, 19]. However, when caregivers fail to acknowledge or support the process of maturation, and key acts of redefining oneself through self-directed change, they can interfere with the trajectory towards emerging adulthood.

## Data Availability

The datasets used and/or analysed during the current study are not publicly available due to confidentiality but are available from the corresponding author on reasonable request.

## Abbreviations

RILS: Residential immersive life skills
TIP: The Independence Program
